# Doxycycline Plus Bortezomib-Containing Regimens for the Treatment of Light-Chain Amyloidosis in the Frontline Setting: Experience from the Amyloidosis Program of Calgary

**DOI:** 10.3390/curroncol31090415

**Published:** 2024-09-18

**Authors:** Ellen Lewis, Nowell Fine, Sylvia McCulloch, Jason Tay, Peter Duggan, Paola Neri, Nizar Bahlis, Victor H. Jimenez-Zepeda

**Affiliations:** 1 Tom Baker Cancer Centre, 1331 29th St., NW, Calgary, AB T2N 4N2, Canada; ellen.lewis@ahs.ca (E.L.); sylvia.mcculloch@ahs.ca (S.M.); jason.tay@ahs.ca (J.T.); peter.duggan@ahs.ca (P.D.); epneri@ucalgary.ca (P.N.); nbahlis@ucalgary.ca (N.B.); 2 Department of Cardiac Sciences, University of Calgary, Calgary, AB T2N 4N2, Canada; nowell.fine@ahs.ca; 3 Arnie Charbonneau Cancer Research Institute, Calgary, AB T2N 4N2, Canada

**Keywords:** light-chain amyloidosis, bortezomib, doxycycline

## Abstract

**Background:** Pre-clinical and retrospective data suggest that doxycycline added to treatment regimens has benefit in AL amyloidosis. However, a recent multicenter, open-label, randomized controlled trial in AL amyloidosis patients treated with CyBorD did not demonstrate a progression-free survival (PFS) or cardiac PFS benefit with added doxycycline. **Objective:** The main objective of this study was to explore the role of doxycycline combined with bortezomib-containing regimens (BCRs) for newly diagnosed AL amyloidosis patients with cardiac involvement and to compare them with a cohort of concurrent patients treated with BCR only. **Material and Methods:** AL amyloidosis patients, newly diagnosed between January 2012 and March 2022, who were treated with BCR at the Amyloidosis Program of Calgary (APC) were evaluated. **Results:** Sixty-four concurrent patients were identified. Thirty-nine patients received doxycycline in addition to BCR (BCR-D) for a median of 8 months. The overall response rate was similar among the groups. No significant differences in VGPR/CR, dFLC at 1 month, time to first response, time to best response, or organ responses were noted between the BCR alone and BCR-D groups. **Summary and Conclusions:** Our retrospective study demonstrated that doxycycline combined with BCR failed to prolong OS, PFS, or cardiac responses compared with BCR alone in patients with cardiac AL amyloidosis.

## 1. Introduction

Light-chain (AL) amyloidosis is a plasma cell disease resulting from the abnormal production of toxic light chains in the bone marrow [1]. These toxic light chains aggregate in the extracellular spaces of organs and tissues, impairing structure and function [1]. AL amyloidosis is a rare disease with an incidence noted to be 10 per million [2,3] and, given this, patients are often diagnosed in later stages with advanced disease [4,5,6]. Treatment for AL amyloidosis therefore needs to be easily tolerated given the fragility of the impacted organs, particularly the heart [6]. Traditionally, treatment for AL amyloidosis involves bortezomib-containing regimens (BCRs) [7,8,9,10]. In the last decade, the antibiotic doxycycline has been studied for its use in light-chain fibril formation and studies have shown mixed efficacy [11,12,13,14,15]. Doxycycline is a derivative of tetracycline, which has been confirmed to have beneficial properties in vitro for several types of amyloidosis. In vitro studies have demonstrated that doxycycline is an inhibitor of matrix metalloproteinases, which is increased in the serum, heart, and kidneys of patients with AL amyloidosis and may be responsible for the organ damage induced by the light-chain deposition process [11]. Additionally, the treatment of isolated light-chain amyloid with doxycycline can directly disrupt the formation of light-chain fibrils and promote the formation of non-cytotoxic aggregates [11,12,13].

The addition of doxycycline has been associated with both providing a mortality benefit [11,12,13,14] and a failure to improve the outcomes of amyloidosis patients [15]. Given the mixed data on the efficacy of doxycycline, our center sought to review our data, spanning a decade, of doxycycline use in our cohorts of AL amyloidosis patients treated with BCR. Our aim is to compare hematologic responses, organ responses, and progression-free and overall response rates between patients treated with BCR alone compared to BCR with doxycycline (BCR-D) via the Amyloidosis Program of Calgary (APC). 

## 2. Background

Initial in vivo studies on doxycycline efficacy demonstrated that doxycycline inhibits amyloid deposition and disrupts the formation of amyloigenic fibrils [13]. Additional rat models demonstrated doxycycline’s possible role as a non-selective inhibitor of matrix metalloproteinase, which, if left uncontrolled, can lead to impaired homeostasis in cardiac myocytes [11]. In vitro models also noted doxycycline’s use in modulating AL autophagy [11]. Given the early positive results seen with the addition of doxycycline, centers around the globe have recommended the use of doxycycline in newly diagnosed AL amyloidosis patients, given at a dose of 100 mg twice daily [9,11,12,13,14]. In 2017, Wechalekar and colleagues published a retrospective review on the addition of doxycycline to BCR regimens in 30 AL amyloidosis patients, compared to 73 matched controls [12]. Wechalekar et al. noted that the addition of doxycycline provided a mortality benefit, which was more pronounced with Mayo stage IIIa and IIIb patients [12]. Moreover, in 2020, D’Souza and colleagues published a phase-2 trial on the impact of doxycycline taken at diagnosis for 1 year, enrolling 25 AL amyloidosis patients, with results showing low overall mortality rates at 1 year and highlighting doxycycline tolerability [14]. In contrast, a more recent multicenter, randomized control trial has put the efficacy of doxycycline into question [15]. Shen and colleagues studied 140 AL amyloidosis patients, with 70 patients randomized to receive doxycycline in addition to BCR, compared to 70 controls. Shen et al. noted that the addition of doxycycline failed to improve progression-free survival (PFS) or enhance cardiac outcomes [15]. Given the mixed results of current studies and the relatively small volume of evidence, the APC undertook a retrospective review. 

## 3. Materials and Methods

This study consisted of a retrospective review of 64 AL amyloidosis patients with cardiac involvement treated with BCR alone (n = 25) compared to a concurrent cohort of BCR-D (n = 39)-treated patients. All patients were seen via the APC and data were collected between the years of January 2012 and March 2022. Doxycycline was added to BCR and given at a standard dose of 100 mg twice daily from the time of initial diagnosis to progression or toxicity. Doxycycline was given as the standard of care at our center starting in the year 2018. Clinical outcomes including hematologic and organ responses were compared between the cohorts and an assessment of responses was performed in accordance with the consensus criteria published in 2005 and modified in 2012 [16,17,18]. All patients who received at least 1 cycle were included.

This study was approved by the Health Research Ethics Boards of Alberta and conducted in accordance with the Declaration of Helsinki. Pertinent clinical data, including bone marrow aspirate/biopsy results, organ function tests, and serum free light-chain assays, were collected.

### 3.1. Study Aims

The main objective of this study was to explore the differences in clinical outcomes between cardiac-involved AL amyloidosis patients treated with BCR-D versus BCR alone. Primary endpoints include hematologic and organ responses, overall survival (OS), and PFS. Secondary endpoints include median dFLC (difference between involved and un-involved free light chains) at 1 month, median time to first response, and median time to best response. 

### 3.2. Treatment Regimes

Doxycycline was added to one of two main BCRs utilized by the APC. The first is CyBorD, which consists of cyclophosphamide, bortezomib, and dexamethasone [7,8]. The second is CyBorMe, which consists of cyclophosphamide, bortezomib, and methylprednisone [19]. Approval for CyBorMe was attained at our center in 2019, after which the majority of treatment-eligible patients received CyBorMe as the standard of care. 

### 3.3. Response Assessment 

Patients included in this study received an AL amyloidosis diagnosis and disease stage based on the international consensus criteria [16,17,18]. Hematologic and organ responses were also monitored utilizing the consensus criteria created in 2005 and updated in 2012 [16,17]. Patients were monitored biochemically and clinically every 2–4 weeks. Median time to initial response was assessed from time of treatment initiation to achievement of partial response (PR). Median time to best response was assessed from time of treatment initiation to best overall response achieved. 

### 3.4. Statistics

Patients’ demographic variables were summarized by appropriate central measures of tendency and dispersion. A two-sided Fisher exact test was used to test for differences between categorical variables. A *p* value of <0.05 was considered significant. A *t*-test was used to compare continuous variables which were normally distributed, and the Wilcoxon rank sum test was used otherwise. A *p* value of <0.05 was considered significant. Survival curves were constructed according to the Kaplan–Meier method and compared using a log-rank test. All statistical analyses were performed using the SPSS 24.0 software.

## 4. Results

A total of 64 concurrent patients with AL amyloidosis and manifested cardiac involvement seen at the APC between January 2012 and March 2022 were included (Table 1). Thirty-nine patients, with a median age of 68 years, received doxycycline in addition to BCR (BCR-D). The median cycles of BCR-D received was eight and a total of five BCR-D-treated patients discontinued doxycycline due to toxicity (7.6% [n = 3] GI toxicity; 5.1% [n = 2] skin toxicity). Twenty-five patients, median age 64, received BCR alone. Most of the patients were lambda light-chain-restricted (76.9% BCR-D vs. 68% BCR; *p* = 0.6; Table 1) and of male predominance (53.8% BCR-D vs. 64% BCR; *p* = 0.4; Table 1). In addition to cardiac involvement, the majority also had kidney involvement (66.6% BCR-D vs. 76% BCR; *p* = 0.4; Table 1). There were no statistically significant differences between cohorts in terms of bone marrow plasma cell concentration or clinical stage; however, a large portion of both cohorts had stage IV disease at diagnosis (43.5% BCR-D vs. 56% BCR; *p* = 0.2; Table 1).

All patients included in this study received first-line BCR with or without the addition of doxycycline. There were baseline differences noted between groups in terms of what specific BCR protocol was utilized. CyBorD was given to 51.2% of patients treated with BCR-D and 80% of patients in the BCR group. More patients in the BCR-D cohort were treated with CyBoMe (48.7% vs. 8%; *p* = 0.001; Table 2) and no patients in the BCR-D cohort were given a clinical trial drug in addition to CyBorD (0% BCR-D vs. 8% BCR; *p* = 0.001; Table 2. This was a non-daratumumab-based trial).

### 4.1. Hematologic Responses

**Figure 1:**
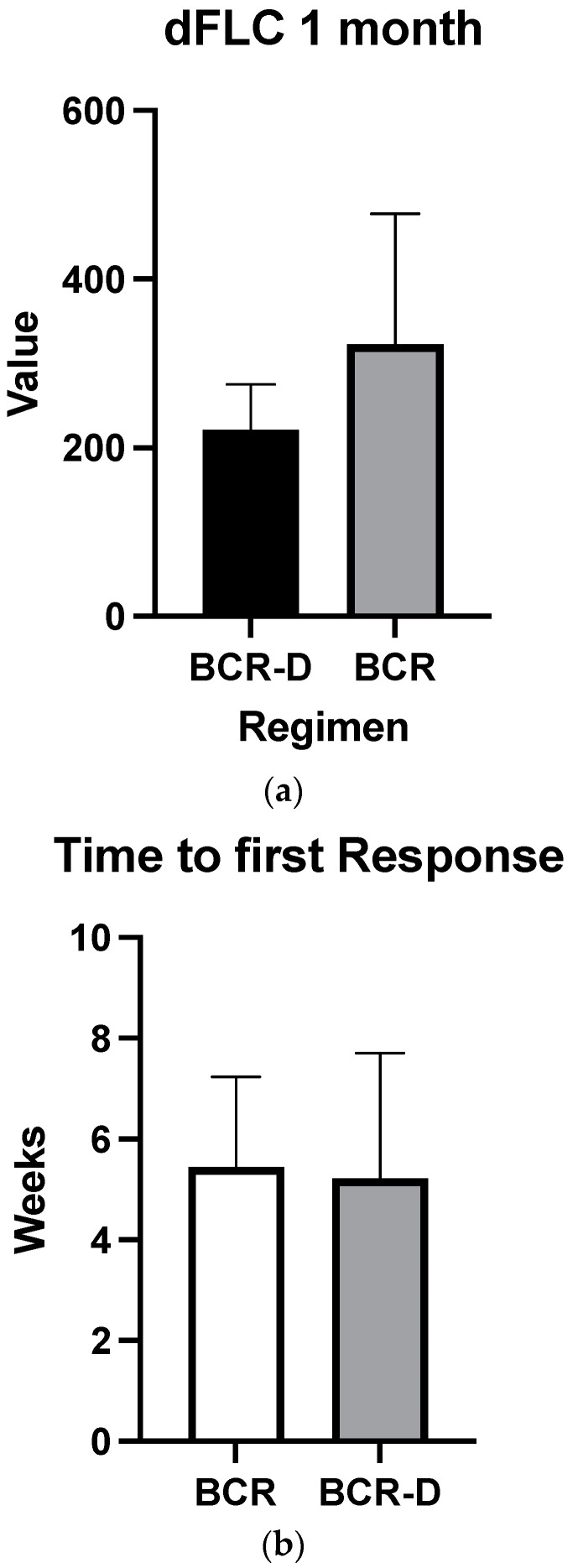

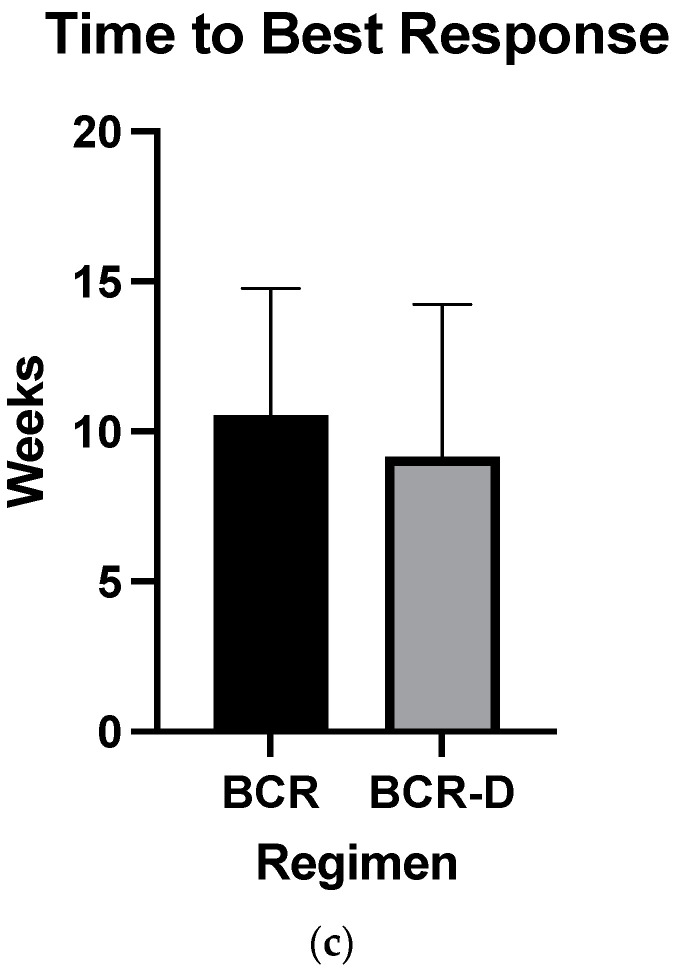
(**a**) Median dFLC at 1 month according to treatment with BCR or BCR-D. Median dFLC at 1 month was not statistically significant between the cohorts (69 mg/L vs. 50.5 mg/L for BCR and BCR-D, respectively; *p* = 0.5). (**b**) Median time to first response according to treatment with BCR or BCR-D. Median time to first response was equal between the cohort (4 weeks; *p* = 0.4). (**c**) Median time to best response according to treatment with BCR or BCR-D. Median time to best response also did not meet statistical significance (12 weeks BCR vs. 8 weeks BCR-D; *p* = 0.2).

Overall hematologic responses were similar between the BCR-D cohort and the BCR-alone group (89.7% BCR-D vs. 84% BCR; *p* = 0.4; Table 2). Additionally, there were no statistically significant differences between CR rates (25.6% BCR-D vs. 20% BCR; *p* = 0.3) and VGPR/CR rates (49% BCR-D vs. 60% BCR; *p* = 0.3) between the cohorts (Table 2). Median dFLC at 1 month was not statistically significant between the cohort (69 mg/L vs. 50.5 mg/L; *p* = 0.5) (Figure 1a). Median time to first response was equal between the cohort (4 weeks; *p* = 0.4) (Figure 1b) and median time to best response also did not meet statistical significance (12 weeks BCR vs. 8 weeks BCR-D; *p* = 0.2) (Figure 1c).

### 4.2. Organ Response

Overall organ responses were seen in 41% (n = 16) of the BCR-D cohort and 48% (n = 12) of the BCR cohort (*p* = 0.5). No statistically significant differences were observed between the two cohorts in terms of cardiac and renal responses. Cardiac responses were seen in 38.4% (15/39) of the BCR-D patients and 56% (14/25) of the BCR patients (*p* = 0.1). Renal responses were seen in 34.6% (9/26) of the BCR-D-treated patients and 47% (9/19) of the BCR patients (*p* = 0.3).

### 4.3. Progression-Free and Overall Survival

**Figure 2:**
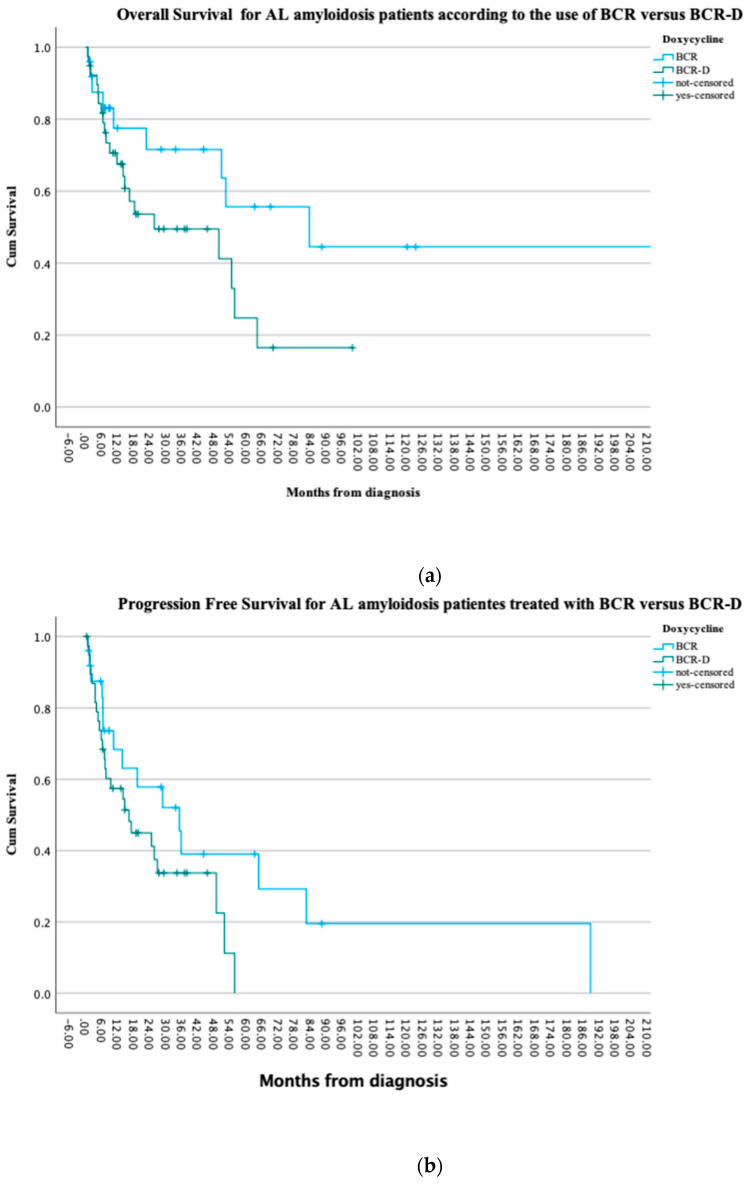
(**a**) Overall survival for AL amyloidosis patients treated at the APC according to the use of BCR and BCR-D. The median OS was not reached in the BCR cohort when compared to a median OS of 25.6 months in the BCR-D cohort (*p* = 0.07). (**b**) Progression-free survival for AL amyloidosis patients treated at the APC according to the use of BCR and BCR-D. There were no statistically significant PFS differences observed between the cohorts (*p* = 0.08).

At the time of this analysis, 41% (n = 16) of the BCR-D and 72% (n = 18) of the BCR patients were alive (*p* = 0.2) and 13 patients in each the BCR-D and BCR cohorts had progressive disease (52% vs. 33.3%; *p* = 0.1). There were no statistically significant PFS differences observed between the cohorts (*p* = 0.08). Median OS was not reached in the BCR cohort when compared to a median OS of 25.6 months in the BCR-D cohort (*p* = 0.07). Survival at 1 year was similar between the cohorts, with 84% (n = 21) in BCR-treated patients compared to 70% (n = 27) in the BCR-D-treated patients (*p* = 0.2) (Figure 2a,b).

## 5. Discussion

As amyloidosis is a rare disease and is often diagnosed in later stages, treatment needs to be effective and tolerable so as to not cause more damage to impacted organs [5,6,10]. Doxycycline, a tetracycline antibiotic, has been studied over the past decade for its use in AL amyloidosis treatment given its low-risk side-effect profile and potential for impacting amyloid fibril formation [11,12,13,14,15]. As suggested in pre-clinical data [11,13], recent retrospective reviews have shown benefit in doxycycline use with advanced AL cardiac amyloidosis patients with statistically significant reduction in cardiac biomarkers and reduced mortality in BCR-D-treated patients [12,14]. Although the pathophysiology of amyloidosis is not fully understood, it has been posited that doxycycline can impact cardiomyocyte damage by altering AL amyloid autophagy [11] and its anti-amylogenic effects [13]. Until 2021, there were no randomized trials studying the addition of doxycycline to BCR for the treatment of AL amyloidosis. Recently, Shen and colleagues (2021) [15] published a multi-site RCT showing no significant differences in cardiac or disease outcomes with the addition of doxycycline. As doxycycline remains the standard of care at many treatment centers, more data are needed to confirm the mixed results of doxycycline efficacy. 

Our long-term retrospective analysis demonstrated that the use of doxycycline in conjunction with BCR failed to improve OS, PFS, and hematologic or organ responses in patients with AL amyloidosis. A trend towards worse OS and PFS was seen in the BCR-D group compared to the BCR-only group; this could just be a reflection of the sample size. As doxycycline is not thought to impact plasma cell production [13], we did not expect significant differences in terms of hematologic responses. Many prior studies have shown benefits in terms of organ, particularly cardiac, responses with the addition of doxycycline [11,12,14]. It was interesting that our analysis failed to reproduce these findings. Many centers have continued using doxycycline despite the mixed results in the literature, as it is thought to be a low-risk drug in comparison to chemotherapeutic agents; however, it is challenging to fully appreciate the possible risks with its long-term use in this population. In our study, doxycycline appeared well-tolerated with only five patients discontinuing due to gastrointestinal and skin toxicity. 

Currently, with the advent of daratumumab, international guidelines recommend a combination of cyclophosphamide, bortezomib, dexamethasone (CyBorD), and daratumumab as first-line therapy for patients newly diagnosed with AL amyloidosis [20]. However, patients with advanced heart disease remain difficult to treat as chemotherapy offers potential limiting toxicity. There currently are two mAbs, birtamimab and Anselamimab (formerly, CAEL-101), under investigation as anti-fibril agents [21,22]. It is hoped that these antibodies will provide direct proof of concept by depleting the deposits of light-chain amyloid fibrils from organs, improving their function; these antibodies are tested in patients with advanced-stage disease and will hopefully provide a novel way of treating patients with AL amyloidosis.

Our analysis did have limitations, including the retrospective nature of data collection and smaller sample size. However, the sample of Southern Alberta patients studied is reflective of the global incidence and prevalence of AL amyloidosis [2]. Additional limitations include the differences in BCR protocols between the patients treated with doxycycline, with the majority of patients receiving CyBorMe. As a note, CyBorMe as utilized at our center has been shown to be well-tolerated and yield quicker hematologic and organ responses when compared to CyBorD [19]; therefore, this is unlikely to be a confounder. Patients on clinical trials were only seen in the BCR-alone group and did not include a daratumumab-based strategy.

## 6. Conclusions

In conclusion, our study noted no statistically significant differences in terms of hematologic responses, organ responses, and progression-free and overall survival between AL amyloidosis patients treated with BCR versus BCR-D. Despite the addition of doxycycline alongside standard-of-care BCR at multiple treatment sites globally [9,14], the evidence appears mixed in terms of its benefits. Our analysis is reflective of the recent RCT conducted by Shen et al., 2021 [15], which showed that doxycycline failed to enhance response and cardiac outcomes for AL amyloidosis patients. These results should encourage further robust research on the use and potential risks of long-term doxycycline use in this patient population. 

## Figures and Tables

**Table 1:** Clinical characteristics of patients with AL amyloidosis receiving BCR according to the use of concomitant doxycycline.

Characteristic	BCR + Doxycycline, n = 39	BCR Alone, n = 25	*p* Value
Age (median)	68	64	0.3
Gender			0.4
Male	21 (53.8%)	16 (64%)	
Female	18 (46.1%)	9 (36%)	
Hb (g/L)	124	122	0.4
Creatinine (µmol/L)	96	80	0.5
B2microglobulin (µmol/L)	3.0	3.24	0.3
Albumin (g/L)	29	32	0.08
Stage I	2 (5.1%)	0 (0%)	0.2
Stage II	4 (10.2%)	4 (16%)	
Stage III	14 (35.8%)	4 (16%)	
Stage IV	17 (43.5%)	14 (56%)	
Unknown	2 (5.1%)	3 (12%)	
LDH (IU/L)	226	232	0.4
BMPC (%)	12	10	0.3
NTproBNP ng/L	2642	3246	0.4
Troponin T ng/L	53	65	0.4
Light chain:			0.6
Kappa	8 (20.5%)	8 (32%)	
Lambda	30 (76.9%)	17 (68%)	
Biclonal	1 (2.5%)	0	
Organ Involvement			
Cardiac involvement	39 (100%)	25 (100%)	NS
Kidney involvement	26 (66.6%)	19 (76%)	0.4
Liver involvement	7 (17.9%)	2 (8%)	0.2
Nerve involvement	4 (10.2%)	5 (20%)	0.2
GI involvement	8 (20.5%)	5 (20%)	0.9
Lung involvement	1 (2.5%)	2 (8%)	0.3

**Table 2:** Treatment regimens and response rates for patients with AL amyloidosis receiving BCR or BCR-D at the Amyloidosis Program of Calgary from 2012 to 2022.

Characteristic	BCR-D, n = 39	BCR, n = 25	*p* Value
**Bortezomib-Containing Regimens**			0.001
CyBorD	20 (51.2%)	20 (80%)	
CyBorMe	19 (48.7%)	2 (8%)	
CyBord plus clinical trial (no daratumumab)	0 (0%)	2 (8%)	
Other	0	1 (4%)	
Median number of cycles of the BCR part	4	6	0.6
**Hematological Response**			
Overall response rate	35 (89.7%)	21 (84%)	0.4
VGPR/CR	19 (49%)	15 (60%)	0.3
Complete response	10 (25.6%)	5 (20%)	0.3
dFLC at 1 month (median)	69	50.5	0.5
Time to first response (median)	4 weeks	4 weeks	0.4
Time to best response (median)	12 weeks	8 weeks	0.2
**Organ Response**			
Overall organ response	16 (41%)	12 (48%)	0.5
Cardiac response	15/39 (38.4%)	14/25 (56%)	0.1
Renal response	9/26 (34.6%)	9/19 (47%)	0.3

## Data Availability

The data are not publicly available for confidential reasons. However, they are secured as per local HREBA policies.
